# Role of Hydrogen Sulfide in the Treatment of Fibrosis

**DOI:** 10.7759/cureus.18088

**Published:** 2021-09-18

**Authors:** Swathy S Chirindoth, Ivan Cancarevic

**Affiliations:** 1 Internal Medicine, California Institute of Behavioral Neurosciences & Psychology, Fairfield, USA

**Keywords:** hydrogen sulfide, fibrosis

## Abstract

Hydrogen sulfide (H_2_S) is a biological gas, the abnormal metabolism of which has associations with the pathogenesis of fibrosis. The purpose of this paper was to determine the potential of H_2_S in the prevention and treatment of fibrosis. The data is obtained mainly from articles found in the PubMed database using the keywords “fibrosis” and “hydrogen sulfide,” limiting the results to those published within the last 10 years. Some additional resources have also been used, such as books and articles within journals. Evidence of decreased H_2_S enzyme levels in animal models with fibrotic diseases has been found. The protective role of H_2_S has been validated by the administration of exogenous H_2_S donors in animal models with fibrosis. It is also evident that H_2_S is involved in complex signaling pathways and ion channels that inhibit fibrosis development. These findings support the role of H_2_S in the treatment of a variety of fibrotic diseases. A randomized controlled trial in fibrosis patients comparing the efficacy of exogenous H_2_S and placebo in addition to standard of care can be implemented to validate this further.

## Introduction and background

Fibrotic diseases are a fatal group of disorders affecting many people worldwide [1]. They affect many organs, including the liver, bone marrow, lung, kidney, gastrointestinal tract, skin, eye, and endocardium. They include systemic fibrotic diseases such as systemic sclerosis and organ-specific disorders, including chronic kidney disease (CKD), liver cirrhosis, idiopathic pulmonary fibrosis, and myocardial fibrosis. The underlying etiologies, as well as the molecular mechanism of many fibrotic diseases, are less clear. They all have complex pathogenetic mechanisms of an uncontrolled and progressive accumulation of fibrotic tissue in affected organs, causing their dysfunction [1]. Epithelial or endothelial damage secondary to irritants (e.g., radiation, chronic infections, toxins) triggers fibrosis’ pathogenesis. Dysfunction of the coagulation cascade and immune system causes chronic production of proteolytic enzymes, fibrogenic cytokines, growth factors, and angiogenic factors, ultimately resulting in fibrosis [2].

Local fibroblasts get attracted to the injury site by platelet-derived growth factor (PDGF), transforming growth factor beta (TGF-β), and tumor necrosis factor alpha (TNF-α) [3]. Later, they differentiate into myofibroblasts, which play a crucial role in fibrogenesis [4]. Myofibroblasts are the activated repair cells needed to restore tissue integrity after injury by producing and organizing the extracellular matrix (ECM) [3,4]. Malfunction of myofibroblasts is considered responsible for excessive synthesis, deposition, and remodeling of extracellular matrix proteins in fibrosis [3,4]. It results in the loss of cellular homeostasis and the disruption of typical tissue architecture, eventually causing organ fibrosis [2]. Currently, anti-fibrotic drugs (e.g., pirfenidone, nintedanib) are the only effective therapy. They are not curative but only manage to slow the rate of progression of common fibrotic diseases, such as idiopathic pulmonary fibrosis [5].

Hydrogen sulfide (H_2_S) is considered the third biological gas after nitrous oxide and carbon monoxide. It acts as a gaso-neurotransmitter. It influences cellular signaling pathways and the sulfhydration of target proteins [6]. H_2_S is produced endogenously by activating two major H_2_S-generating enzymes (cystathionine β-synthase (CBS) and cystathionine γ-lyase (CSE)) [7]. It involves many physiological processes in the cardiovascular, neuronal, immune, respiratory, gastrointestinal, liver, and endocrine systems. It also has potent properties, such as inhibiting inflammation and oxidative stress in several organs [7,8]. Abnormal metabolism of H_2_S causes damage to the structure and function of many organs [7]. All the above findings lead us to propose the association of H_2_S with fibrosis pathogenesis and the potential to help treat fibrosis.

Methodology

In this review, data from clinical trials and reviews relevant to the role of H_2_S in the treatment of fibrosis are displayed. The idea of this study is to reveal the role of H_2_S in the prevention and treatment of fibrosis. The articles used in this review were found in the PubMed database using the keywords “fibrosis” and “hydrogen sulfide.” Studies published more than 10 years ago were excluded. Some additional resources have also been used, such as books and articles within journals. Due to limited access to some articles, a full quality assessment of individual articles could not be performed.

## Review

Hydrogen sulfide in renal fibrosis

Renal fibrosis is characterized by renal parenchymal cell injury causing interstitial inflammatory cell infiltration, fibroblast proliferation, and myofibroblast transformation, which leads to excessive extracellular matrix (ECM) deposition and fibrogenesis [9,10]. This progressive disease leads to chronic kidney disease, a common global health challenge [11]. H_2_S plays a physiological role in maintaining the kidney’s normal function and is found in discrete parts of the kidney [7,12]. Dugbartey discussed the protective mechanisms of H_2_S in experimental models of chronic kidney disease (CKD) [11]. H_2_S under hypoxic conditions acts as an oxygen sensor in the renal medulla and restores oxygen balance [11]. It also increases medullary flow [11]. Han et al. revealed the experimental data showing H_2_S deficiency leading to renal fibrosis [13]. Wang et al. demonstrated that H_2_S stimulates tubular cell regeneration, reduction in apoptosis, autophagy, and hypertrophy, which improved vascular remodeling and high blood pressure [14]. According to Qian et al., the reduction of endogenous H_2_S contributes to diabetic injury [15]. The supply of exogenous H2S protects tissues from diabetic damage through anti-apoptosis and anti-fibrosis mechanisms, inhibited oxidative stress, and inflammation [15]. Sun et al. provided evidence from experimental studies suggesting the involvement of the H_2_S signaling pathway in the treatment of diabetic nephropathy [16]. 

Hydrogen sulfide donor molecules, such as GYY4137, have led to significant decreases in inflammation, fibrosis, and the expression of epithelial-mesenchymal transition markers following urinary obstruction [17]. Sodium hydrosulfide (product of the half-neutralization of H_2_S with sodium hydroxide) protects against unilateral ureteral obstruction (UUO)-induced renal damage by attenuating fibrosis, oxidative stress, and inflammation [18]. H_2_S also exerts a preventive effect on UUO-induced kidney damage in rats by reducing oxidative stress [19].

There is a reduction in the levels of H_2_S production by renal cells in pathological conditions, including diabetic nephropathy [15,16]. The benefits of H_2_S donors in kidney injury have been evident [15]. In CKD patients and animal models, plasma H_2_S level has been reported to be markedly reduced [12]. Aging-induced kidney changes have been shown to alleviate following H_2_S administration, probably by inhibiting signaling pathways leading to matrix protein synthesis [20]. It exhibits potent antioxidant, anti-inflammatory, and anti-fibrotic properties in several experimental kidney disease models [12]. It also suppresses inflammation and oxidative stress, inhibiting activation of fibrosis-related cells and cytokine expression [14]. These findings suggest that H_2_S and its transsulfuration pathway may be a potential target for developing therapeutics for fibrosis-related diseases [21].

Hydrogen sulfide in hepatic fibrosis

Diverse stimuli, such as ethanol, viral infection, and toxins, can cause hepatic fibrosis. The activated hepatic stellate cells (HSCs) transform into myofibroblast-like cells. These cells can proliferate and produce extracellular matrix, leading to the destruction of the architecture of the liver parenchyma and cirrhosis [12]. The liver helps maintain plasma H_2_S homeostasis by regulating its production and elimination [7]. Three endogenous H_2_S-producing enzymes (cystathionine γ-lyase (CSE), cystathionine β-synthase (CBS), and 3-mercaptopyruvate sulfurtransferase (MST)) are all present in the liver. CSE and CBS play a significant role in hepatic H_2_S production. In human hepatic fibrosis and cirrhosis, as well as in animal and cellular models of hepatic fibrosis, the metabolic levels of H_2_S and its producing enzymes were recorded to change [12]. Mani et al. found that in many liver diseases, such as hepatic fibrosis and hepatic cirrhosis, malfunction of hepatic H_2_S metabolism may be involved [22]. Fan et al. validated that exogenous H_2_S inhibits proliferation and induces cell cycle arrest and apoptosis in activated hepatic stellate cells (HSCs) and attenuates carbon tetrachloride (CCl_4_)-induced hepatic fibrosis and ECM gene expression [23]. Zhang et al. displayed hindrance of pro-fibrogenic properties and reduced oxidative stress by H_2_S-associated mechanism in HSCs [24].

The results mentioned above support the involvement of endogenous H_2_S in the pathogenesis of human and animal hepatic fibrosis. These findings suggest that H_2_S may be implicated in hepatic fibrosis, and the modulation of H_2_S production may represent a therapeutic remedy for liver fibrosis. To further prove the protective role of H_2_S in hepatic fibrosis, more research is needed.

Hydrogen sulfide in myocardial fibrosis

Any mechanical stretch or inflammatory stimuli can cause the proliferation and activation of cardiac fibroblast resulting in cardiac fibrosis. It is associated with the excessive formation of extracellular matrix within the myocardium [25,26]. Locally released angiotensin II can stimulate cardiac fibroblast proliferation and increase collagen production by activating the angiotensin II type1 (AT1) receptor [27]. Aldosterone can also induce cardiac fibrosis by exhibiting pro-inflammatory effects and directly promoting cardiac fibroblast proliferation and collagen synthesis [28]. Interstitial fibrosis is a partial manifestation of cardiac remodeling. It eventually leads to chronic heart failure [12]. H_2_S could protect against endoplasmic reticulum stress-induced endothelial-to-mesenchymal transition through the Src pathway [29]. There is evidence of H_2_S causing a reduction in fibronectin and galectin‐3, the inhibition of which prevents cardiac remodeling by interfering with myocardial fibrogenesis [30]. It also inhibits the local renin-angiotensin-aldosterone system (RAAS) [12]. H_2_S exhibits angiogenic and anti-inflammatory actions. It also preserves mitochondrial function and reduces apoptosis [31]. H_2_S suppresses potassium channel (K^+^ channel) activity, attenuating atrial fibroblast proliferation and differentiation toward myofibroblasts [32]. GYY4137, a slow-releasing H_2_S donor, causes enhanced early postischemic endogenous natriuretic peptide activation [33]. It preserves cardiac function and attenuates adverse remodeling [33]. It may also exert postischemic cardioprotective effects, such as pro-angiogenesis, anti-apoptosis, anti-hypertrophy, and anti-fibrosis [33]. Meng et al. also supported the protective role of GYY4137 in myocardial fibrosis, which is by inhibiting oxidative stress, blocking the TGF-β1/Smad2 signaling pathway, and decreasing the expression of α-smooth muscle actin (α-SMA) [34]. Pan et al. provided strong evidence that exogenous H_2_S prevented cardiac remodeling through ECM accumulation inhibition and increased vascular density [35].

Endogenous H_2_S and its producing enzymes were involved in cardiac fibrosis development [36-40]. In different animal models of cardiac fibrosis, endogenous H_2_S levels and H_2_S enzyme expressions were reduced [36-40]. Exogenous H_2_S has demonstrated a protective effect in various animal models of cardiac fibrosis [32-34,41]. Liposomal formulations, which release H_2_S slowly within tissues, have exhibited enhanced cardioprotective effects in vivo via the inhibition of the TGF-β1/Smad signaling pathway [41]. Overall, all the above studies have shown the protective role of H_2_S in myocardial fibrosis.

Hydrogen sulfide in pulmonary fibrosis

Idiopathic pulmonary fibrosis is one of the most common interstitial lung diseases with debilitating dyspnoea symptoms and cough. Scarring of alveolar walls, airways, or vasculature causes irreversible lung function impairment. The significant derangement of ventilatory function and gas exchange contributes to chronic respiratory insufficiency with a severely impaired quality of life, ultimately leading to respiratory failure and death. It is a fatal disease with limited effective therapeutic options. H_2_S is produced endogenously in the respiratory system, and it targets epithelial cells, fibroblasts, airway, and pulmonary artery smooth muscle cells [42]. A decrease in H_2_S-producing enzymes and endogenous H_2_S levels is associated with pulmonary fibrosis development [7]. The inhibition of proliferation, migration, and differentiation of human lung fibroblasts have shown protective effects against pulmonary fibrosis [12]. The metabolism of H_2_S in the lungs may serve as a biomarker for specific respiratory diseases [42]. Hence, it may prove useful for the prevention and treatment of selective chronic respiratory diseases, including fibrosis.

The studies mentioned above show that endogenous H_2_S in the respiratory tract regulates essential functions, such as airway tone, pulmonary circulation, cell proliferation or apoptosis, fibrosis, oxidative stress, and inflammation [43]. Chronic inflammation can result from high concentrations of H_2_S (50-500 ppm), which can lead to pulmonary fibrosis [8].

**Figure 1:**
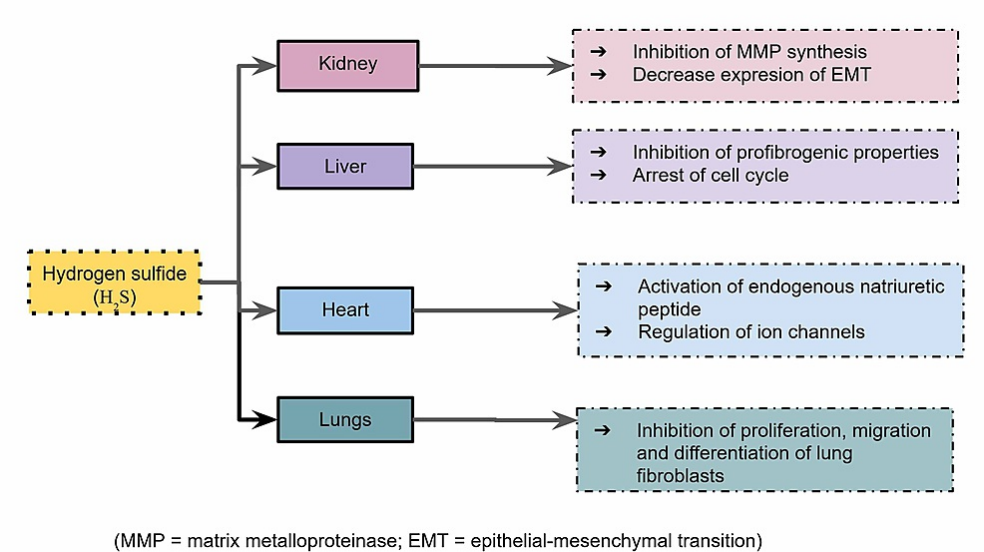
The main mechanisms of hydrogen sulfide in different organs preventing fibrosis MMP, matrix metalloproteinase; EMT, epithelial–mesenchymal transition

The mechanisms of H_2_S in preventing fibrosis of different organs are listed in Figure 1. The abovementioned observations from the articles included in this review are shown in Table 1. Table 2 presents the comparison of current treatments of fibrosis with H2S.

**Table 1:** Brief observation of articles included in this review H_2_S, hydrogen sulfide; UUO, unilateral ureteral obstruction; HSCs, hepatic stellate cells; CCl_4_, carbon tetrachloride; ECM, extracellular matrix; EMT, endothelial-to-mesenchymal transition; α-SMA, α-smooth muscle actin; RAAS, renin–angiotensin–aldosterone system

Author	Conclusion
Renal fibrosis	
Dugbartey [11]	H_2_S restores oxygen mechanism and increases medullary blood flow.
Han et al. [13]	H_2_S deficiency leads to fibrosis.
Wang et al. [14]	H_2_S resulted in improved vascular remodeling and high blood pressure.
Qian et al. [15]	Reduced endogenous H_2_S contributes to diabetic injury; exogenous H_2_S protects tissues from diabetic damage.
Sun et al. [16]	The H_2_S signaling pathway is involved in the treatment of diabetic nephropathy.
Lin et al. [17]	H_2_S donor molecules led to significant decreases in inflammation, fibrosis, and the expression of epithelial–mesenchymal transition markers following urinary obstruction.
Jiang et al. [18]	Sodium hydrosulfide protects against UUO-induced renal damage.
Dursun et al. [19]	H_2_S has preventive effect on UUO-induced kidney damage in rats by reducing oxidative stress.
Lee et al. [20]	Aging-induced kidney changes are alleviated following H_2_S administration by inhibiting signaling pathways leading to matrix protein synthesis.
Hepatic fibrosis	
Song et al. [12]	The metabolic levels of H_2_S and its producing enzymes were recorded to change in hepatic fibrosis.
Mani et al. [22]	Hepatic H_2_S metabolism’s malfunction may be involved in many liver diseases.
Fan et al. [23]	Exogenous H_2_S inhibits proliferation and induces cell cycle arrest and apoptosis in activated HSCs and attenuates CCl_4_-induced hepatic fibrosis and ECM gene expression.
Zhang et al. [24]	Diallyl trisulfide hinders pro-fibrogenic properties and reduces oxidative stress by an H_2_S-associated mechanism in HSCs.
Myocardial fibrosis	
Ying et al. [29]	H_2_S could protect against endoplasmic reticulum stress-induced EMT through the Src pathway.
Snijder et al. [30]	H_2_S causes a reduction in fibronectin and galectin‐3, the inhibition of which prevents cardiac remodeling by interfering with myocardial fibrogenesis.
Shen et al. [31]	H_2_S exhibits angiogenic and anti-inflammatory actions; it also preserves mitochondrial function and reduces apoptosis.
Sheng et al. [32]	H_2_S suppresses potassium channel activity, attenuating atrial fibroblast proliferation and differentiation toward myofibroblasts.
Lilyanna et al. [33]	GYY4137, a slow-releasing H_2_S donor, cause enhanced early postischemic endogenous natriuretic peptide activation; it may also exert postischemic cardioprotective effects.
Meng et al. [34]	GYY4137 inhibits oxidative stress, blocks the TGF-β1/Smad2 signaling pathway, and decreases the expression of α-SMA.
Pan et al. [35]	Exogenous H_2_S prevented cardiac remodeling through ECM accumulation inhibition and increased vascular density.
Tran et al. [41]	Liposomal formulations, which release H_2_S slowly within tissues, have exhibited enhanced cardioprotective effects in vivo via the inhibition of the TGF-β1/Smad signaling pathway.
Song et al. [12]	H_2_S inhibits the local RAAS.
Pulmonary fibrosis	
Chen et al. [42]	H_2_S metabolism in the lungs is associated with respiratory diseases.
Zhang et al. [7]	A decrease in H_2_S-producing enzymes and endogenous H_2_S levels is associated with pulmonary fibrosis development.
Bazhanov et al. [43]	Endogenous H_2_S in the respiratory tract regulates essential functions.

**Table 2:** Comparison of current treatments of fibrosis with hydrogen sulfide RAAS, renin–angiotensin–aldosterone system; ET_A_R, endothelin A receptor; TGF-β, transforming growth factor beta; SGLT2, sodium–glucose transport protein 2; H_2_S, hydrogen sulfide; EMT, epithelial–mesenchymal transition; ECM, extracellular matrix; HSCs, hepatic stellate cells

Fibrosis	Current treatments	Possible role of H_2_S
Renal	RAAS blocker, ET_A_R antagonist, TGF-β inhibitor, inflammation modulators, SGLT2 inhibitor	Exogenous H_2_S can protect the kidneys by inhibiting signaling pathways leading to matrix protein synthesis [20]. H_2_S donor molecules led to significant decreases in inflammation, fibrosis, and the expression of EMT markers [17].
Myocardial	RAAS inhibitors, inflammation modulators, TGF-β inhibitors, endothelin inhibitors, β-blockers, ivabradine, loop diuretics, sildenafil	Exogenous H_2_S prevents cardiac remodeling through ECM accumulation inhibition and increased vascular density [35]. Liposomal formulations, which release H_2_S slowly within tissues, have exhibited enhanced cardioprotective effects in vivo via the inhibition of the TGF-β1/Smad signaling pathway [41]. GYY4137, a H_2_S donor, can cause enhanced early postischemic endogenous natriuretic peptide activation. It may also exert postischemic cardioprotective effects [33].
Hepatic	Treating the underlying disorder, liver transplantation for patients with advanced stages of liver fibrosis	Exogenous H_2_S inhibits proliferation and induces cell cycle arrest and apoptosis in activated hepatic stellate cells [23]. It also hinders pro-fibrogenic properties and reduces oxidative stress by H_2_S-associated mechanisms in HSCs [24].
Pulmonary	Nintedanib and pirfenidone are associated with very high morbidity and mortality	Endogenous H_2_S in the respiratory tract regulates essential functions [43]. The association of H_2_S metabolism in lungs with respiratory diseases can be used to prevent this from occurring [42].

## Conclusions

In this review, the role of H_2_S in fibrotic diseases, a major fatal irreversible group of disorders for which no current treatment has proved effective in stopping the progression, is discussed. It has been demonstrated that H_2_S plays a protective role in developing fibrosis in the lung, liver, kidney, and heart. This protective effect in fibrosis has been established by the administration of exogenous H_2_S donors in animal models. H_2_S inhibits fibrosis development by involving targeted signaling pathways and suppressing the K^+^ channel activity. These results imply that the H_2_S-producing enzymes or H_2_S itself might be a potential therapeutic target for fibrosis. To better understand the potential role of H_2_S in the treatment of fibrotic disorders, a randomized controlled trial comparing the efficacy of H_2_S and placebo in addition to the current standard of care should be performed.
